# Interleukine-6 in critically ill COVID-19 patients: A retrospective analysis

**DOI:** 10.1371/journal.pone.0244628

**Published:** 2020-12-31

**Authors:** Julie Gorham, Anthony Moreau, Francis Corazza, Lorenzo Peluso, Fanny Ponthieux, Marta Talamonti, Antonio Izzi, Carole Nagant, Narcisse Ndieugnou Djangang, Alessandra Garufi, Jacques Creteur, Fabio Silvio Taccone

**Affiliations:** 1 Department of Intensive Care, Erasme Hospital, Université Libre de Bruxelles (ULB), Brussels, Belgium; 2 Department of Immunology, Laboratoire Hospitalier Universitaire de Bruxelles (LHUB-ULB), Translational Research, Campus Horta, ULB, Brussels, Belgium; Azienda Ospedaliero Universitaria Careggi, ITALY

## Abstract

**Introduction:**

Coronavirus disease 2019 (COVID-19) appeared in China in December 2019 and has spread around the world. High Interleukin-6 (IL-6) levels in COVID-19 patients suggest that a cytokine storm may play a major role in the pathophysiology and are considered as a relevant parameter in predicting most severe course of disease.

The aim of this study was to assess repeated IL-6 levels in critically ill COVID-19 patients admitted to our Intensive Care Unit (ICU) and to evaluate their relationship with patient’s severity and outcome.

**Methods:**

We conducted a retrospective study on patients admitted to the ICU with a diagnosis of COVID-19 between March 10 (i.e. the date of the first admitted patients) and April 30, 2020. Demographic, clinical and laboratory data were collected at admission. On the day of IL-6 blood concentration measurement, we also collected results of D-Dimers, C-Reactive Protein, white blood cells and lymphocytes count, lactate dehydrogenase (LDH) and ferritin as well as microbiological samples, whenever present.

**Results:**

Of a total of 65 patients with COVID-19 admitted to our ICU we included 41 patients with repeated measure of IL-6. There was a significant difference in IL-6 levels between survivors and non-survivors over time (p = 0.001); moreover, non survivors had a significantly higher IL-6 maximal value when compared to survivors (720 [349–2116] vs. 336 [195–646] pg/mL, p = 0.01). The IL-6 maximal value had a significant predictive value of ICU mortality (AUROC 0.73 [95% CI 0.57–0.89]; p = 0.01).

**Conclusions:**

Repeated measurements of IL-6 can help clinicians in identifying critically ill COVID-19 patients with the highest risk of poor prognosis.

## Introduction

Since December 2019, a virus named “severe acute respiratory syndrome coronavirus 2” (SARS-CoV-2) appeared in Wuhan, China, and has since spread rapidly around the world [1]. Disease caused by SARS-CoV-2 has been identified as “coronavirus disease 2019” (COVID-19) by the World Health Organization (WHO). The majority of patients presenting COVID-19 develop mild to moderate symptoms, with a quick recovery; however, patients who develop severe forms have a high mortality rate, which can reach up to 60% in patients admitted to the Intensive Care Unit (ICU) and treated with mechanical ventilation [2].

Some studies have suggested that lymphopenia and cytokine release syndrome (CRS) are associated with the severity of the disease [2,3]; CRS is a systemic inflammatory response that can be triggered by a variety of factors, such as infections, toxins or an idiosyncratic response to medications, and is characterized by an increased level of pro-inflammatory cytokines, including interleukin-6 (IL-6). This so-called “cytokine storm” can rapidly cause single or multiple organ failure and has been considered to determine not only the severity but also the prognosis of COVID-19 [4]. Indeed, several studies have shown higher than normal IL-6 levels in COVID-19 patients requiring hospitalization or presenting with acute respiratory failure [5–7]. The results of a recent meta-analysis also showed that patients with complicated forms of COVID-19 (i.e. ICU admission and/or acute respiratory failure) had nearly three-fold higher serum IL-6 levels than those with non-complicated disease [8]. High levels of IL-6 were also significantly related to some clinical features, such as the maximum body temperature or the presence of bilateral pulmonary involvement on chest computed tomography [7]. Finally, higher IL-6 levels were observed amongst non-survivors from COVID-19 when compared to those who survived [4].

As these data suggest that IL-6 level could serve as an indicator of poor prognosis, some authors have considered IL-6 as an important target for COVID-19 therapeutics. Indeed, tocilizumab, a recombinant humanized monoclonal anti-IL-6 receptor antibody, has already been incorporated into the Chinese and Italian COVID-19 management guidelines, although the results of ongoing clinical trials should be awaited to better define its role in this setting.

The aim of this study was therefore to assess repeated IL-6 levels in critically ill COVID-19 patients admitted to the ICU and to evaluate their relationship with patient’s severity and outcome. Also, we evaluated whether available biological biomarkers could be helpful to predict high IL-6 levels in this setting.

## Methods

### Study design

This was a single-center retrospective analysis conducted in the Department of Intensive Care of the Hôpital Erasme in Brussels, Belgium, between March 10 (i.e. the date of the first admitted patients) and April 30, 2020. The study protocol was approved by the local ethics committee (P2020/251) and the informed written consent was waived for the retrospective design of the study.

### Study population

Patients admitted in the ICU were eligible for the study if: 1) they were adult (≥ 18 years of age); 2) had a confirmed diagnosis of COVID-19 with real-time polymerase chain reaction (rRT-PCR) assay on nasopharyngeal swab and/or broncho-alveolar lavage (BAL) specimens for SARS-CoV-2; 3) IL-6 measurement was decided by the treating physician.

### Patients’ management

Patients diagnosed with COVID-19 were admitted to the ICU according to the severity of lung impairment and the requirement for non-invasive or invasive mechanical ventilation. Medical treatment was decided according to the treating physician and the Infectious Diseases specialist. In general, patients received oral hydroxychloroquine (400 mg q12h orally on the first day followed by 200 mg q12h orally until day 10), which was associated with an antiviral drug (i.e. remdesivir, lopinavir-ritonavir or oseltamivir), according to drug availability.

### Data collection

Demographics, medical history, clinical data and main biological findings on the day of admission were collected from the electronic medical file. On the day of IL-6 blood concentration measurement, we also collected results of D-Dimers, C-Reactive Protein, white blood cells and lymphocytes count, lactate dehydrogenase (LDH) and ferritin as well as microbiological samples, whenever present. Data on ventilatory parameters (i.e. tidal volume, minute ventilation, positive end-expiratory pressure–PEEP, FiO_2_ and PaO_2_/FiO_2_) and on the hemodynamic status (i.e. lactate, total and cardiovascular Sequential Organ Failure Assessment score) [9] were also collected. We also recorded the daily use of specific therapies, such as vasopressors, corticosteroids, prone positioning, the use of renal replacement therapy (RRT) or extra-corporeal membrane oxygenation (ECMO). Mortality was recorded at ICU discharge. Laboratory testing for IL-6 became available at the end of March 2020; routine IL-6 measurements were then performed for ICU patients on Monday and Thursday of each week. The IL-6 blood concentration was measured using an ELISA method from BD Biosciences (BD OptEIA Human IL-6 ELISA Kit II, San Jose, CA). All tests were performed according to the manufactory’s instructions. Serum samples were collected in serum separator tubes, centrifuged and stored at -20°C until assessment.

### Outcomes of the study

The primary outcome was the proportion of IL-6 measurements exceeding 1000 pg/mL; this cut-off has been used to treat septic shock patients with endotoxin hemoadsorption therapy [10]. A maximum of four IL-6 measurements per patients were collected. Secondary outcomes included: a) correlations between IL-6 and other available biological variables; b) the association of the daily value with patients’ condition (i.e. use of ECMO and/or prone positioning, diagnosis of a secondary infection), severity (i.e. PaO_2_/FiO_2_ <100 or SOFA > 8); c) time-course of IL-6 levels over time between survivors and non-survivors as well as difference between the first or the highest IL-6 values between these two groups (i.e. association of IL-6 with mortality).

### Statistical analysis

Descriptive statistics were computed for all study variables. A Kolmogorov-Smirnov test was used and histograms and normal-quartile plots were examined to verify the normality of distribution of continuous variables. Data are presented as count (percentage), mean (± standard deviation) or median [25th–75th percentiles], as appropriate. Differences between subgroups were assessed using a χ-square or Fisher’s exact test for categorical variables, as appropriate, and a t-test or a Mann Whitney-U test for continuous variables, at each time-point. Correlation between IL-6 and other biological biomarkers will be assessed using Spearman correlation. The differences in IL-6 levels between survivors and non-survivors was analyzed using a generalized mixed model. The discriminative ability of the maximal highest IL-6 value to predict ICU mortality was evaluated using receiver operating characteristic (ROC) curves with the corresponding area under the curve (AUROC). Youden’s index was computed to assess the optimal cut-off of the highest IL-6 value for sensitivity and specificity to predict ICU mortality. A p<0.05 will be considered as statistically significant. Statistical analyses will be performed using IBM SPSS Statistics 25 for Macintosh.

## Results

### Study population

A total of 65 patients were admitted to the ICU over the study period with confirmed COVID-19; of those, 10 patients were admitted before the IL-6 assessment was routinely available at the laboratory, 10 showed a rapidly improvement of their clinical status and were discharged without IL-6 assessment and 4 were not tested for IL-6. The characteristics of the study cohort, including 41 patients, are shown in Table 1; median age was 58 [53–64] years and the most common comorbidity was arterial hypertension (n = 23, 56%). Overall, 35 (85%) patients were treated with mechanical ventilation, 28 (68%) with prone positioning, 13 (32%) with RRT and 8 (20%) with ECMO. Thirteen (32%) patients died during the ICU stay.

**Table 1:** Characteristics of the study population. Data are presented as median [IQRs] or count (percentage).

	n = 41
Male gender, n (%)	32 (78)
Age, years	58 [53–64]
***COMORBIDITIES***	
Hypertension, n (%)	23 (56)
Ischemic Heart Disease, n (%)	6 (15)
COPD, n (%)	12 (29)
Diabetes, n (%)	6 (15)
Chronic Renal Failure, n (%)	2 (5)
Liver Cirrhosis, n (%)	2 (5)
Immunosuppression, n (%)	6 (15)
Cancer, n (%)	4 (10)
Previous Cerebrovascular Disease, n (%)	-
Smoking, n (%)	4 (10)
Obesity, n (%)	17 (41)
Previous DVT, n (%)	-
***DIAGNOSIS***	
rRT-PCR positive on swab	39 (95)
rRT-PCR positive on BAL ^#^	19 (83)
***ON ADMISSION***	
Temperature on admission, °C	37.5 [37.0–38.2]
WBC on admission, n/mm^3^	9120 [6070–12160]
Lymphocytes on admission n/mm^3^	750 [610–950]
Hemoglobin admission, g/dL	13 [13–14]
Platelets on admission, n*10^3^/mm^3^	241 [147–290]
C-Reactive Protein on admission, mg/dL	180 [120–240]
D-Dimers on admission, ng/mL	1902 [1208–3989]
LDH on admission, IU/L	498 [410–631]
hs-Troponin I on admission, ng/L	21 [13–39]
PaO_2_/FiO_2_ on admission	132 [95–156]
***DURING THE ICU STAY***	
Non-invasive ventilation, n (%)	20 (49)
Mechanical Ventilation, n (%)	35 (85)
Prone Positioning, n (%)	28 (68)
ECMO, n (%)	8 (20)
Vasopressors, n (%)	29 (71)
Acute Kidney Injury, n (%)	23 (56)
Renal Replacement Therapy, n (%)	13 (32)
Hydroxychloroquine, n (%)	35 (85)
Remdesivir, n (%)	3 (7)
Lopinavir/Ritonavir, n (%)	21 (51)
Oseltamivir, n (%)	5 (12)
***OUTCOMES***	
Tracheostomy, n (%)	8 (20)
ICU death, n (%)	11 (27)
ICU discharge, n (%)	9 (22)

^#^ Total broncho-alveolar lavage (BAL) = 23.

COPD = chronic obstructive pulmonary disease; DVT = deep venous thrombosis; rRT-PCR = reverse real time polymerase chain reaction; WBC = while blood cells; LDH = lactate dehydrogenase; hs = high sensitivity; ECMO = extra-corporeal membrane oxygenation; ICU = intensive care unit.

### IL-6 measurements

The first measurement of IL-6 was performed after a median of 4 [2–7] days after ICU admission; 34 (83%) patients had a second measurement after 7 [6–11] days, 30 (73%) a third measurement after 11 [10–15] days and 25 (61%) after 16 [13–20] days, respectively, for a total of 130 measurements. For each time-point, median IL-6 values were 314 [164–440] pg/mL, 188 [102–588] pg/mL, 133 [64–209] pg/mL and 133 [107–182] pg/mL, respectively; the highest IL-6 value per patient was 406 [265–730] pg/mL. A total of 11 (8%) measurements exceeded 1000 pg/mL; 4/41 (10%) on the first measurement, 3/34 (9%) on the second, 3/30 (10%) on the third and 1/25 (4%) on the fourth.

### IL-6 and other biological variables

The correlation coefficients between IL-6 and the other biological data are shown in Table 2. IL-6 was significantly correlated to CRP only on the first (r = 0.37; p 0.02) and the third measurement (r = 0.63; p <0.01). Also, we observed a correlation between IL-6 and white blood cells count on the third measurement (r = 0.47; p<0.01), between IL-6 and D-Dimers on the forth measurement (r = 0.45; p = 0.03), between IL-6 and LDH on the second measurement (r = 0.37; p = 0.03) and between IL-6 and lactate on the first measurement (r = 0.31; p = 0.05). No other correlation were observed with other available biological variables.

**Table 2:** Correlation between IL-6 and different biochemical variables.

	MEASURE 1	MEASURE 2	MEASURE 3	MEASURE 4
	***r***	***r***	***r***	***r***
	***P value***	***P value***	***P value***	***P value***
Platelets, n*10^3^/mm^3^	0.04	- 0.17	- 0.20	- 0.33
	0.80	0.34	0.29	0.11
PaO_2_/FiO_2_	0.16	- 0.08	0.02	- 0.22
	0.31	0.65	0.93	0.30
White blood cells, n/mm^3^	0.12	0.09	0.47	0.01
	0.47	0.62	<0.01	0.95
Lymphocytes, n/mm^3^	0.02	- 0.21	0.19	0.04
	0.91	0.23	0.32	0.85
C-reactive protein, mg/L	0.37	0.28	0.63	0.32
	0.02	0.11	<0.01	0.12
Ferritine, mcg/L	0.23	- 0.01	- 0.21	0.19
	0.14	0.97	0.28	0.37
D-Dimers, ng/mL	- 0.30	- 0.13	0.00	0.45
	0.06	0.45	0.98	0.03
LDH, IU/L	0.11	0.37	0.04	0.18
	0.49	0.03	0.84	0.40
Lactate, mmol/L	0.31	0.20	0.31	0.12
	0.05	0.26	0.10	0.59

LDH = lactate dehydrogenase.

### IL-6 and in different subgroups

IL-6 levels in different subgroups of patients are summarized in Table 3. Patients treated with ECMO had higher IL-6 values than other patients on the second and third measurement. Also, patients treated with corticosteroids had significantly lower IL-6 levels than others on the second measurement, despite similar values on the first measurement. No other significant differences between subgroups were observed.

**Table 3:** IL-6 levels in different subgroups of patients.

	MEASURE 1	MEASURE 2	MEASURE 3	MEASURE 4
ECMO	228 (133–382)	1013 (319–1821)	216 (146–462)	134 (119–150)
	n = 6	n = 6	n = 7	n = 4
No ECMO	329 (183–451)	160 (89–329)	101 (60–175)	133 (107–182)
	n = 35	n = 29	n = 23	n = 21
	p = 0.38	p = 0.04	p = 0.048	p = 0.92
ECMO and/or PP	400 (164–500)	426 (133–768)	175 (68–269)	134 (127–179)
	n = 17	n = 14	n = 12	n = 6
No ECMO/PP	954 (646–3029)	135 (89–286)	115 (63–189)	133 (92–175)
	n = 24	n = 21	n = 18	n = 19
	p = 0.53	p = 0.13	p = 0.44	p = 0.64
Corticosteroids	370 (164–793)	49 (28–71)	23 (22–68)	131 (98–147)
	n = 4	n = 4	n = 4	n = 4
No Corticosteroids	314 (164–412)	194 (131–627)	140 (73–214)	133 (107–193)
	n = 37	n = 31	n = 26	n = 21
	p = 0.85	p <0.01	p = 0.08	p = 0.63
Infection	390 (166–558)	178 (99–650)	140 (96–321)	136 (105–160)
	n = 16	n = 18	n = 10	n = 7
No Infection	310 (154–388)	188 (115–329)	98 (61–202)	130 (108–187)
	n = 25	n = 17	n = 20	n = 18
	p = 0.26	p = 0.59	p = 0.37	p = 0.79
PaO_2_/FiO_2_ < 100	291 (159–390)	487 (173–1798)	106 (36–189)	2158
	n = 11	n = 4	n = 6	n = 1
PaO_2_/FiO_2_ ≥ 100	332 (175–490)	188 (88–458)	140 (64–211)	n = 133 (100–171)
	n = 30	n = 32	n = 24	n = 23
	p = 0.42	p = 0.20	p = 0.60	p = 0.11
SOFA < 8	330 (199–401)	160 (115–638)	63 (34–92)	134 (110–251)
	n = 12	n = 9	n = 6	n = 20
SOFA ≥8	314 (159–437)	190 (97–519)	151 (80–255)	133 (21–162)
	n = 28	n = 26	n = 24	n = 5
	p = 0.94	p = 0.75	p = 0.13	p = 0.38

ECMO = extra-corporeal membrane oxygenation; PP = prone positioning; SOFA = Sequential Organ Failure Assessment.

### IL-6 and outcome

**Fig 1:**
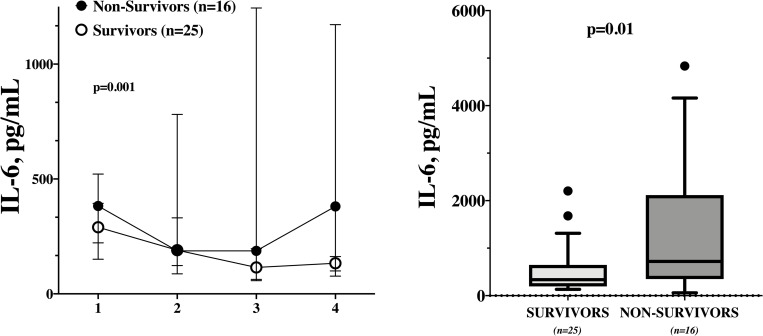
IL-6 values over time in survivors and non-survivors (LEFT); differences in the highest IL-6 value in survivors and non-survivors (RIGHT).

There was a significant difference in IL-6 levels between survivors and non-survivors over time (Fig 1; p = 0.001); non-survivors had also a significantly higher IL-6 maximal value when compared to non-survivors (720 [349–2116] vs. 336 [195–646] pg/mL, p = 0.01 –Fig 1). The IL-6 maximal value had a significant predictive value of ICU mortality (AUROC 0.73 [95% CIs 0.57–0.89]; p = 0.01). The Youden’s index identified the threshold of IL-6 > 406 pg/mL for the best combination of sensitivity (75%) and specificity (64%) to predict ICU mortality.

## Discussion

This study focused on IL-6 values in critically ill COVID-19 patients admitted to the ICU. The main findings were: a) IL-6 blood concentrations were high but exceeded 1000 pg/mL in less than 10% of measurements; b) the correlation of IL-6 with other available biological variables was relatively poor so IL-6 levels cannot be extrapolated from other variables in this setting; c) higher IL-6 levels were observed in ECMO patients when compared to others, but no other critical conditions, including multiple organ failure and hypoxemia, was associated with high IL-6 levels; d) the maximal IL-6 value over the ICU stay was associated with ICU mortality.

Several studies have reported IL-6 levels in COVID-19 patients; results of most of those have been summarized in S1 Table. Most of the existing studies showed that IL-6 levels were higher than normal ranges in COVID-19 patients [6,11,12]; also, IL-6 levels were greater in severe cases of COVID-19 than in mild to moderate forms [13,14], although it was unclear whether these cases corresponded to patients admitted to the ICU or just those suffering from moderate to severe lung involvement. Moreover, the number of severe/ICU patients was relatively small in all these studies. The number (i.e. once vs. multiple) as well as timing of IL-6 measurement (i.e. on admission vs. during hospital/ICU stay) were also different among studies, which limited the comparison with our findings. The lack of information on the assay methods for IL-6 and the poor analytic standardization would be also significant confounders for data comparison. Our study specifically focused on ICU patients with a high level of severity (i.e. high SOFA score; 85% of patients on mechanical ventilation, 68% treated with prone positioning and 20% with ECMO), which resulted in an overall mortality of 32%; as such, our results are representative of a severe subgroup of critically ill COVID-19 patients with a high risk of poor outcome. Larger cohorts of ICU COVID-19 patients with multiple IL-6 assessments would be necessary to more specifically assess the significance and the prognostic role of this cytokine in this setting.

High IL-6 levels in COVID-19 patients have been considered as the most objective demonstration of the ongoing “cytokine storm”, as it has been observed in patients with septic shock, cardiac surgery or those receiving treatment with chimeric antigen receptor–transduced T cells (CAR-T) against acute leukemia [15–17]. IL-6 can exert its action on all cell types by binding the transmembrane (cis-signaling) or soluble form (trans-signaling) of the IL-6 receptor (IL-6R) and forming a complex with gp130 that activates its Janus kinase (JAK) effector. The activation of several downstream pathways would result in various biological effects, such as the maturation of naïve T cells into effector T cells, the expression of vascular endothelial growth factor (VEGF) in endothelial cells, the increase in vascular permeability and reduction of myocardial contractility, which contribute to organ damage and increased risk of death in COVID-19 patients [18]. Indeed, the first COVID-19 *post-mortem* study reported extensive alveolar edema with macrophages and monocyte infiltrations of the lung and a decrease in CD4+ and CD8+ cells and an increase in Th-17 cells, which were related to high IL-6 levels [19]. Our findings, however, raise some concerns about the concept of “cytokine storm” during COVID-19. First of all, COVID-19 patients do not have all the features, such as capillary leakage, distributive shock, disseminated intravascular coagulation, that are observed in sepsis, cardiac surgery or CAR-T syndrome [15–17]. Second, IL-6 levels were far lower than those observed in septic shock, where median values ranged around 5000 pg/mL [20]; also critically ill patients with hyperinflammatory status or those undergoing open heart operation with subsequent hyperdynamic circulatory instability and organ dysfunction often exceeded IL-6 levels of 5000 pg/mL [21]. According to the selected threshold [10], our study suggests that IL-6 levels were certainly higher than normal values in critically ill COVID-19 patients but definitely lower than other overwhelming acute inflammatory syndromes with multiple organ failure. Whether the association of high IL-6 with poor outcome is just a marker of severity or a potential target for specific IL-6 blocking therapies, it remains to be better studies. As it has been reported that the severity of pulmonary immune injury correlates with extensive infiltration of neutrophils and macrophages in the lung tissues [22], it could be interesting to assess IL-6 levels into the bronchoalveolar lavage (BAL), as high IL-6 levels have already been reported in BAL of viral H7N9 pneumonia patients [23]. A better understanding on cytokine levels and immune reaction during COVID-19 would be then necessary to understand how to select patients who might benefit from specific therapies, such as IL-6 pathways blockers or corticosteroids.

In our study, we found an association between IL-6 and CRP in critically COVID-19 patients; this finding was already reported in other studies [24,25] and is related to the effects of IL-6 and other cytokines on hepatic synthesis of inflammatory proteins, such as CRP [26]. However, this correlation was weak, as some other sporadic correlations of IL-6 levels with other biological variables, such as white blood cells count, D-dimers, LDH and lactate. Our findings could be biased by the small sample size of the study or by the inclusion of critically ill patients having predominantly high IL-6 levels. Indeed, a correlation between IL-6 and CRP is often observed within lower ranges of IL-6 values (i.e. 50–300 pg/mL) [27]. Importantly, the methods of IL-6 measurement were probably different among studies (i.e. different analytical methods or total vs. free IL-6 values assessment), which would limit the reproducibility and comparison of the overall findings. In view of our results, using CRP or any other biological variable to identify patients with high IL-6 levels seems to be unreliable.

Most of previous studies showed an association of high IL-6 levels with the progression of COVID-19 severity and with poor outcome [28–30]. In our study, we observed a different time-course significant of IL-6 levels between survivors and non-survivors, although the small sample size and the different timing for sampling could have limited the consistency of the analysis. Also, we observed a higher maximal IL-6 value in non-survivors than in survivors; this result suggests that the disease might have different inflammatory trajectories during the ICU stay and repeated IL-6 measurements could be more relevant than one single measurement to assess patients’ severity in this setting. It could be higher in specific subgroups such as those treated with ECMO. Other subgroups with a higher inflammatory profile could be better identified in larger studies.

Our study has some limitations. First, it is a retrospective single-center study with the biases inherent of this type of study. Second, because of the limited number of patients, it is difficult to assess risk factors for elevated IL-6 levels using a multivariable adjusted analysis. Third, some of these patients experienced secondary superinfection, which might be a significant confounder to evaluate the association of IL-6 with COVID-19 severity. Forth, lack of correlation between IL-6 and other biological variables in samples taken after several days of ICU stay might be influenced by other confounders, which are independent from COVID-19. Fifth, IL-6 levels were not measured at standard intervals; also, patients with mild COVID-19 forms were not included, which may have influenced the accuracy of IL-6 to predict severity and/or outcomes. Sixth, other studies have also reported that IL-6 during COVID-19 are similar if not lower than in patients suffering from sepsis or cardiac arrest [31,32]; however, we performed repeated IL-6 samplings and provided a time-course assessment in critically ill ICU patients, which was missing in these previous studies. Finally, IL-6 is only of the cytokines involved in the pathogenesis of severe forms of COVID-19; whether multiple cytokine rather than IL-6 alone assessment might be more appropriate to evaluate the “cytokine storm” in these patients, it should be clarified in future studies.

In conclusions, only a minority of ICU patients presented very high IL-6 levels during COVID-19. Repeated measurements of IL-6 can help the clinicians in identifying critically COVID-19 patients with the highest risk of poor prognosis.

## Supporting information

S1 Table Summary of studies reporting IL-6 levels in COVID-19 patients. * severe patients instead of critically ill patients admitted into the Intensive Care Unit (ICU).
(DOCX)
