# Supplementary material for: Interleukine-6 in critically ill COVID-19 patients: A retrospective analysis
Source: PLoS One. 2020 Dec 31;15(12):e0244628. doi: 10.1371/journal.pone.0244628 (PMC7774924; doi:10.1371/journal.pone.0244628)
Supplement: S1 Table — * severe patients instead of critically ill patients admitted into the Intensive Care Unit (ICU). (DOCX) [file pone.0244628.s001.docx]

**S1 Table.** Summary of studies reporting IL-6 levels in COVID-19 patients. * severe patients instead of critically ill patients admitted into the Intensive Care Unit (ICU).

| **Study** | **Type of study** | **N** | **N ICU** | **Measurements of IL-6** | **Timing of IL-6** | **Methods of IL-6 measurement** | **Median IL-6 values**  **in ICU/severe patients** | Main results |
| --- | --- | --- | --- | --- | --- | --- | --- | --- |
| **Cai et al** | R | 298 | 30 | Multiple | Admission and  then routinely | NR | 26.95 pg/mL | Significantly higher IL-6 levels in severe vs. mild patients  High level of IL-6 was an independent risk-factor for the development of severe COVID-19 forms |
| **Chen et al** | P | 29 | 5 | NR | NR | NR | 72 pg/ml* | Significantly higher IL6 levels in critical ill patients vs. others  Significantly higher IL6 levels in severe vs. mild patients |
| **Chen et al** | R | 99 | 23 | Once | Admission | NR | NR | Levels of IL- 6 over normal ranges in COVID-19 patients |
| **Chen et al** | R | 48 | 17 | Once | Admission | ELISA | NR | Significantly higher IL-6 levels in patients with viremia vs. others  Significantly higher IL-6 levels in non-survivors vs. survivors |
| **Chen et al** | R | 21 | 11* | NR | NR | NR | NR | Significant higher levels of IL-6 in severe vs non severe patients |
| **Chen et al** | R | 548 | 48 | Multiple | Routinely | NR | 8.33pg/ml | Significant higher Il-6 admission levels in survivors vs non survivors  Progressive increase of IL-6 levels is a risk factor for fatal outcomes |
| **Diao et al** | R | 522 | 43 | Multiple | Routinely | Flow cytometry | 186 pg/mL | Significantly higher IL-6 levels in ICU vs. non ICU patients |
| **Gao et al** | R | 43 | 15* | Once | Admission | Immunofluorescence | 36.10 pg/mL* | High level of IL-6 was an independent risk-factor for the development of severe COVID-19 forms |
| **Herold et al** | R | 40 | 40 | Multiple | Routinely | Elecsys system immunoassay | 37pg/ml | Circulating levels of Il-6 is highly predictive of the need for invasive ventilation  Elevated Il-6 levels in the course of the disease predicted significantly respiratory failure |
| **Hou et al** | R | 389 | 52 | Twice | Admission and at the time of disease aggravation | Automatic procedure of a solid-phase two-site chemiluminescent immunometric assay | NR | Il-6 levels significantly increased with increased severity of illness |
| **Huang et al** | P | 41 | 13 | Once | Admission | NR | 6.1 pg/mL | Significantly higher IL-6 levels in ICU vs. non ICU patients |
| **Latif et al** | R | 28 | 7 | Multiple | Admission and  then routinely | NR | NR | High IL- 6 levels in 88% of patients (i.e. heart transplant) |
| **Li et al** | R | 54 | 23 | Once | Within 24h from admission | NR | < 300 pg/mL | Significantly higher IL-6 levels in ICU vs. non-ICU patients |
| **Li et al** | R | 548 | 269* | Once | Admission | NR | NR | Significant higher IL-6 levels in severe patients vs non severe |
| **Liu et al** | R | 140 | 33* | Once | Within 24h from admission | NR | NR | Significantly higher IL-6 levels in severe vs. non-severe patients |
| **Liu et al** | R | 80 | 3 | Multiple | Admission and  then routinely | ELISA | 36.6 pg/ml* | Correlation between IL-6 levels were correlated with disease severity  Correlation between IL-6 levels and CRP, LDH, ferritin and D-dimers  IL-6 levels were influenced by therapies (i.e. steroids, polyvalent IGs, HFOT and MV Significantly reduced IL-6 levels after treatment together with improved CT-scan findings |
| **Liu et al** | R | 64 | 0 | Once | Admission | NR | NR | High IL-6 levels on admission was an unfavorable factor for hospital discharge |
| **Liu et al** | R | 40 | 13* | Multiple | Routinely | Human Th 1/2 cytosine kit II | < 200 pg/ml | Higher serum IL-6 levels in patients without methylprednisolone treatment vs with methylprednisolone treatment  More significant fluctuation in the serum levels serum levels of Il-6 in severe patients vs in mild  Reductions in serum IL-6 levels in the severe group started at 16 days after disease onset |
| **Liu et al** | R | 76 | 30* | NR | NR | NR | 29.1pg/ml* | Significant higher IL-6 levels in the severe group vs in the mild group |
| **Luo et al** | R | 15 | 7 | 8 times | Before and after TCZ | NR | 88 pg/mL | Gradually reduction of IL-6 levels after TCZ |
| **Qin et al** | R | 452 | 286* | Once | Admission | NR | 25.2 pg/mL * | Significantly higher levels of IL- 6 levels in severe vs. non-severe patients |
| **Quartuccio et al** | R | 111 | 27* | Once | Admission | Electrochemiluminescence immunoassay | 63.5pg/ml* | Significant higher level of IL-6 levels in severe cases |
| **Ranucci et al** | P | 16 | 16 | Once | Admission | Quantra Hemostasis | 218 pg/mL | Correlation between IL6 and Fibrinogen levels |
| **Ruan et al** | R | 150 | 41 | Once | Admission | NR | NR | Significantly higher IL-6 levels in non-survivors vs. survivors |
| **Toniati et al** | P | 100 | 46 | 3 times | Admission  Before TCZ  10 days after TCZ | NR | NR | Ten days after TCZ administration, IL-6 levels increased both in improved and worsened patients |
| **Wan et al** | P | 123 | 21* | Twice | First day  1-3 days before discharge | Immunofluorescence | 21 pg/mL* | Significant higher IL-6 levels in severe patients vs mild  Higher survival rates in the mild group and in patients with IL-6 within normal values |
| **Wang et al** | R | 165 | 54* | Multiple | Routinely | NR | 16.6 pg/mL* | Correlation between IL-6 levels and pathological chest CT-scan findings |
| **Wang et al** | P | 65 | 35* | Once | After Admission | Electrochemiluminescence method | NR | Significant higher IL-6 levels in severe patients vs mild |
| **Wang et al** | R | 45 | 12 | Once | Admission | NR | <300pg/ml | Significant higher IL-6 levels in critical group than in severe |
| **Wu et al** | R | 201 | 53 | Multiple | Routinely | NR | NR | Association between ARDS development and high IL-6 levels |
| **Wu et al** | R | 188 | 50 | Once | Admission | NR | NR | Significantly higher IL-6 levels in patients with acute cardiac injury vs. others |
| **Yan et al** | R | 193 | 193* | Once | Admission | NR | NR | Significant higher IL-6 levels in patients with severe Covid-19 patients with diabetes |
| **Yang et al** | R | 52 | 19* | Once | Admission | NR | 33.7 pg/mL * | Significantly higher IL-6 levels severe/critical cancer patients vs mild |
| **Yang et al** | R | 55 | 1 | Multiple | Admission and  then routinely | NR | NR | Significantly higher IL-6 levels in COVID-19 patients with pneumonia vs. others High level of IL-6 was a risk-factor for the development of severe COVID-19 forms  Significantly higher IL-6 levels in non-survivors vs. survivors |
| **Zhang et al** | R | 1 | 1 | Multiple | Routinely | Flow cytometry | < 251.8pg/ml | Patient’s initial higher levels of IL-6 decreased rapidly when the viral RNA was returned as negative |
| **Zhang et al** | R | 111 | NR | Once | Admission | NR | 35.7pg/ml in deterioration group | IL-6 concentrations in deteriorated patients were elevated compared with the discharged patients |
| **Zheng et al** | R | 34 | 34 | Multiple | Routinely | Elisa Method | 47pg/ml | Progressive decrease in IL-6 levels in NIV cases vs IMV cases during hospitalization, and higher levels in IMV cases than in NIV cases |
| **Zhou et al** | R | 191 | 50 | Multiple | Routinely | NR | NR | Significantly higher IL-6 levels in survivors vs. non-survivors |
| **Zhou et al** | R | 21 | 13 | Once | Admission | Automatic biochemical | 17.16 pg/mL | High IL-6 levels in severe patients |
| **Zhu et al** | R | 127 | 3 | Multiple | Routinely | Flow cytometry | NR | High level of IL-6 was an independent risk-factor for the development of severe COVID-19 forms |

1. Cai Q, Huang D, Ou P, Yu H, Zhu Z, Xia Z,et al.COVID-19 in a designated infectious diseases hospital outside Hubei Province, China.Allergy. 2020. doi: 10.1111/all.14309.
2. Chen L, Liu HG, Liu W, Liu J, Liu K, Shang J, et al. [Analysis of clinical features of 29 patients with 2019 novel coronavirus pneumonia]. Zhonghua Jie He He Hu Xi Za Zhi. 2020;43(0):E005.
3. Chen N, Zhou M, Dong X, Qu J, Gong F, Han Y, et al. Epidemiological and clinical characteristics of 99 cases of 2019 novel coronavirus pneumonia in Wuhan, China: a descriptive study. Lancet. 2020;395(10223):507–13.
4. Chen X, Zhao B, Qu Y, Chen Y, Xiong J, Feng Y,et al.Detectable serum SARS-CoV-2 viral load (RNAaemia) is closely correlated with drastically elevated interleukin 6 (IL-6) level in critically ill COVID-19 patients. Clin Infect Dis. 2020.
5. Chen G, Wu D, Guo W, Cao Y, Huang D, Wang H, et al. Clinical and immunological features of severe and moderate coronavirus disease 2019. J Clin Invest. 2020 May;130(5):2620-2629.
6. Chen R, Sang L, Jiang M, Yang Z, Jia N, Fu W, et al. Medical Treatment Expert Group for COVID-19. Longitudinal hematologic and immunologic variations associated with the progression of COVID-19 patients in China. J Allergy Clin Immunol. 2020 May 11:S0091-6749(20)30638-2.
7. Diao B, Wang C, Tan Y, Chen X, Liu Y, Ning L, et al. Reduction and Functional Exhaustion of T Cells in Patients with Coronavirus Disease 2019 (COVID-19). medRxiv. 2020 ;2020.02.18.20024364.
8. Gao Y, Li T, Han M, Li X, Wu D, Xu Y, et al. Diagnostic Utility of Clinical Laboratory Data Determinations for Patients with the Severe COVID-19. J Med Virol 2020, <http://dx.doi.org/10.1002/jmv.25770>.
9. Herold T, Jurinovic V, Arnreich C, Lipworth BJ, Hellmuth JC, von Bergwelt- Baildon M, et al. Elevated levels of interleukin-6 and CRP predict the need for mechanical ventilation in COVID-19. J Allergy Clin Immunol. 2020 May 18.
10. Hou H, Zhang B, Huang H, Luo Y, Wu S, Tang G,et al. Using IL-2R/lymphocytes for predicting the clinical progression of patients with COVID-19. Clin Exp Immunol. 2020 May 4.
11. Huang C, Wang Y, Li X, Ren L, Zhao J, Hu Y, et al. Clinical features of patients infected with 2019 novel coronavirus in Wuhan, China. The Lancet. 2020;395(10223):497–506.
12. Latif F, Farr MA, Clerkin KJ, Habal MV, Takeda K, Naka Y, et al. Characteristics and Outcomes of Recipients of Heart Transplant With Coronavirus Disease 2019.JAMA Cardiol. 2020. doi: 10.1001/jamacardio.2020.2159.
13. Li Y, Hu Y, Yu J, Ma T. Retrospective analysis of laboratory testing in 54 patients with severe- or critical-type 2019 novel coronavirus pneumonia. Lab Invest. 2020. doi: 10.1038/s41374-020-0431-6.
14. Li X, Xu S, Yu M, Wang K, Tao Y, Zhou Y, et al. Risk factors for severity and mortality in adult COVID-19 inpatients in Wuhan. J Allergy Clin Immunol. 2020 Apr 12:S0091-6749(20)30495-4.
15. Liu F, Li L, Xu M, Wu J, Luo D, Zhu Y, et al. Prognostic value of interleukin-6, C-reactive protein, and procalcitonin in patients with COVID-19. J Clin Virol. 2020; 127:104370.
16. Liu T, Zhang J, Yang Y, Ma H, Li Z, Zhang J, et al. The potential role of IL-6 in monitoring severe case of coronavirus disease 2019. medRxiv. 2020 ;2020.03.01.20029769.
17. Liu J, Ouyang L, Guo P, Wu HS, Fu P, Chen YL, et al. Epidemiological, clinical characteristics and outcome of medical staff infected with COVID- 19 in Wuhan, China: A retrospective case series analysis. bioRxiv 2020, <http://dx.doi.org/10.1101/2020.03.09.20033118>.
18. Liu J, T, Zhang J, Yang Y, Ma H, Li Z, Zhang J, et al. The potential role of IL-6 in monitoring severe case of coronavirus disease 2019. medRxiv. 2020 ;2020.03.01.20029769.
19. Liu J, Li S, Liu J, Liang B, Wang X, Wang H, et al. Longitudinal characteristics of lymphocyte responses and cytokine profiles in the peripheral blood of SARS-CoV-2 infected patients. EBioMedicine. 2020 Apr 18;55:102763.
20. Liu Y, Liao W, Wan L, Xiang T, Zhang W. Correlation Between Relative Nasopharyngeal Virus RNA Load and Lymphocyte Count Disease Severity in Patients with COVID-19. Viral Immunol. 2020 Apr 10.
21. Qin C, Zhou L, Hu Z, Zhang S, Yang S, Tao Y, et al. Dysregulation of immune response in patients with COVID-19 in Wuhan, China. Clin Infect Dis. 2020
22. Quartuccio L, Sonaglia A, McGonagle D, Fabris M, Peghin M, Pecori D, et al. Profiling COVID-19 pneumonia progressing into the cytokine storm syndrome: results from a single Italian Centre study on tocilizumab versus standard of care. J Clin Virol. 2020 May 15:104444.
23. Ranucci M, Ballotta A, Di Dedda U, Bayshnikova E, Dei Poli M, Resta M, et al.The procoagulant pattern of patients with COVID-19 acute respiratory distress syndrome.J Thromb Haemost. 2020. doi: 10.1111/jth.14854.
24. Ruan Q, Yang K, Wang W, Jiang L, Song J. Clinical predictors of mortality due to COVID-19 based on an analysis of data of 150 patients from Wuhan, China. Intensive Care Med. 2020
25. Toniati P, Piva S, Cattalini M, Garrafa E, Regola F, Castelli F, et al. Tocilizumab for the Treatment of Severe COVID-19 Pneumonia With Hyperinflammatory Syndrome and Acute Respiratory Failure: A Single Center Study of 100 Patients in Brescia, Italy. Autoimmun Rev.2020;102568.
26. Wan S, Yi Q, Fan S, Lv J, Zhang X, Guo L et al.Relationships among lymphocyte subsets, cytokines, and the pulmonary inflammation index in coronavirus (COVID-19) infected patients.Br J Haematol. 2020 ;189(3):428-437.
27. Wang H, Luo S, Shen Y, Li M, Zhang Z, Dong Y, et al. Multiple enzyme release, inflammation storm and hypercoagulability are prominent indicators for disease progression in COVID-19: a multi-centered, correlation study with CT imaging score. SSRN 2020, <http://dx.doi.org/10.2139/ssrn.3544837>.
28. Wang F, Hou H, Luo Y, Tang G, Wu S, Huang M,et al. The laboratory tests and host immunity of COVID-19 patients with different severity of illness. JCI Insight. 2020 Apr 23:137799.
29. Wang C, Fei D, Li X, Zhao M, Yu K. IL-6 may be a good biomarker for earlier detection of COVID-19 progression. Intensive Care Med. 2020 May 8:1–2.
30. Wu C, Chen X, Cai Y, Xia J, Zhou X, Xu S, et al. Risk Factors Associated With Acute Respiratory Distress Syndrome and Death in Patients With Coronavirus Disease 2019 Pneumonia in Wuhan, China. JAMA Intern Med. 2020
31. Wu C, Hu X, Song J, Du C ,Xu J, Yang D ,et al .Heart injury signs are associated with higher and earlier mortality in coronavirus disease 2019 (COVID-19). bioRxiv 2020, <http://dx.doi.org/10.1101/2020.02.26.20028589>.
32. Yan Y, Yang Y, Wang F, Ren H, Zhang S, Shi X, et al. Clinical characteristics and outcomes of patients with severe covid-19 with diabetes. BMJ Open Diabetes Res Care. 2020 Apr;8(1):e001343.
33. Yang F, Shi S, Zhu J, Shi J, Dai K, Chen X. Clinical characteristics and outcomes of cancer patients with COVID-19.J Med Virol. 2020 doi: 10.1002/jmv.25972.
34. Yang P, Ding Y, Xu Z,Pu R,Li P,Yan J,et al. Epidemiological and clinical features of COVID-19 patients with and without pneumonia in Beijing, China. bioRxiv 2020, <http://dx.doi.org/10.1101/2020.02.28.20028068>
35. Zhang S, Gan J, Chen BG, Zheng D, Zhang JG, Lin RH,et al. Dynamics of peripheral immune cells and their HLA-G and receptor expressions in a patient suffering from critical COVID-19 pneumonia to convalescence. Clin Transl Immunology. 2020 May 10;9(5):e1128.
36. Zhang J, Yu M, Tong S, Liu LY, Tang LV. Predictive factors for disease progression in hospitalized patients with coronavirus disease 2019 in Wuhan, China. J Clin Virol. 2020 Apr 28;127:104392
37. Zheng Y, Sun LJ, Xu M, Pan J, Zhang YT, Fang XL, et al. Clinical characteristics of 34 COVID-19 patients admitted to intensive care unit in Hangzhou, China. J Zhejiang Univ Sci B. 2020 May;21(5):378-387.
38. Zhou F, Yu T, Du R, Fan G, Liu Y, Liu Z, et al. Clinical course and risk factors for mortality of adult inpatients with COVID-19 in Wuhan, China: a retrospective cohort study. Lancet. 2020. https://doi.org/10.1016/S0140- 6736(20)30566-3.
39. Zhou Y, Han T, Chen J, Hou C, Hua L, He S,et al.Clinical and Autoimmune Characteristics of Severe and Critical Cases of COVID-19. Clin Transl Sci. 2020. doi: 10.1111/cts.12805.
40. Zhu Z, Cai T, Fan L, Lou K, Hua X, Huang Z, Gao G. Clinical value of immune-inflammatory parameters to assess the severity of coronavirus disease 2019. Int J Infect Dis. 2020 Apr 22; 95:332-39
